# The Efficacy and Safety of Folate Receptor α‐Targeted Antibody‐Drug Conjugate Therapy in Patients With High‐Grade Epithelial Ovarian, Primary Peritoneal, or Fallopian Tube Cancers: A Systematic Review and Meta‐Analysis

**DOI:** 10.1002/cam4.70392

**Published:** 2024-11-11

**Authors:** Eun Taeg Kim, Ji Hyun Kim, Eun Young Park, In Hye Song, Han Song Park, Sang‐Yoon Park, Myong Cheol Lim

**Affiliations:** ^1^ Department of Obstetrics and Gynecology Kosin University College of Medicine Pusan Republic of Korea; ^2^ Center for Gynecologic Cancer National Cancer Center Goyang Republic of Korea; ^3^ Biostatistics Collaboration Team Research Core Center, National Cancer Center Goyang Republic of Korea; ^4^ Department of Obstetrics and Gynecology Seoul National University College of Medicine Seoul Republic of Korea; ^5^ Cancer Control and Policy, National Cancer Center Graduate School of Cancer Science and Policy National Cancer Center Goyang Republic of Korea; ^6^ Rare & Paediatric Cancer Branch and Immuno‐Oncology Branch, Division of Rare and Refractory Cancer Research Institute, National Cancer Center Goyang Republic of Korea

**Keywords:** adverse event, antibody‐drug conjugate, folate receptor alpha, mirvetuximab soravtansine, ovarian cancer

## Abstract

**Background:**

Antibody‐drug conjugates (ADC) have emerged as a highly promising systemic option in the treatment of recurrent ovarian cancer. The present study aimed to evaluate the treatment efficacy of folate receptor α (FRα)‐targeting ADCs, associated treatment‐related adverse events (TRAEs), and their impact on treatment safety.

**Methods:**

We conducted an electronic search to identify prospective trials of single‐agent ADCs targeting FRα and those combined with chemotherapy in recurrent ovarian cancer. Information regarding the objective response rate (ORR) and TRAEs was collectively analyzed, and differences in subgroups based on FRα receptor expression levels were investigated. The protocol was registered with PROSPERO (CRD42023491151).

**Results:**

Ten studies with a total of 940 patients (859 treated with Mirvetuximab soravtansine‐gynx (MIRV)), 45 with Farletuzumab Ecteribulin (MORAb‐202), and 36 with Luveltamab Tazevibulin (STRO‐002) were included in this meta‐analysis. Based on the pooled data, the ORR of the entire cohort was 37% (95% CI: 0.30–0.43), while that of the high‐FRα expression group was 34% (95% CI: 0.26–0.42). The incidence of grade ≥ 3 adverse events was 27% (95% CI: 0.19–0.36).

**Conclusion:**

FRα‐targeting ADCs, including MIRV, demonstrated definite efficacy and good safety as novel choices for second‐line and beyond treatment of advanced or recurrent ovarian cancer. Patients with high FRα expression showed ORR and PFS benefits similar to those in the overall cohort.

## 1 Introduction

Ovarian cancer remains one of the most lethal gynecologic malignancies, with an estimated 19,710 new cases and 13,270 deaths reported in the US in 2023 [1]. In 2020, an estimated 313,959 new cases of ovarian cancer and 207,252 ovarian cancer‐related deaths were reported worldwide [2]. Epithelial ovarian cancer (EOC), which constitutes approximately 90% of malignant ovarian tumors, initially responds to cytoreductive surgery (CRS) and postoperative systemic treatment with a combination of taxane and platinum cytotoxic chemotherapies [3]. In addition, the development of antiangiogenic drugs and poly (ADP‐ribose) polymerase (PARP) inhibitors as maintenance treatments has fundamentally altered the management of the disease, leading to a significant increase in survival rates [4, 5, 6, 7, 8, 9]. Nevertheless, most cases of advanced ovarian cancer experience recurrence, leading to resistance against systemic therapy and treatment failure [10]. Recent advancements in targeted therapies have begun to transform ovarian cancer into a manageable chronic condition, offering new hope for sustained disease control [11].

Folate receptor alpha (FRα) is a glycosylphosphatidylinositol‐anchored protein that is minimally expressed in normal tissues but is overexpressed in more than 80% of serous ovarian cancer [12]. This overexpression persists despite chemotherapy, highlighting FRα as an attractive therapeutic target [13]. Antibody‐drug conjugates (ADCs) represent a novel therapeutic approach that combines the specificity of monoclonal antibodies with the potent cytotoxic effects of chemotherapeutic agents [12, 14, 15]. By targeting FRα, ADCs can deliver cytotoxic drugs directly to cancer cells, minimizing damage to normal tissues and enhancing therapeutic efficacy [16].

Mirvetuximab soravtansine (MIRV) is one of the leading ADCs targeting FRα, consisting of a humanized anti‐FRα monoclonal antibody linked to the cytotoxic agent DM4 [17]. Clinical studies, including the SORAYA trial, have shown that MIRV provides significant antitumor activity with an acceptable safety profile in patients with recurrent ovarian cancer, leading to its accelerated FDA approval on November 14, 2022, for adults with FRα‐positive, platinum‐resistant ovarian cancer who had previously received one to three systemic treatments [18, 19]. Additionally, other FRα‐targeting ADCs, such as luveltamab tazevibulin (STRO‐002) and farletuzumab ecteribulin (MORAb‐202), have been investigated and demonstrated promising early results [12]. The MIRASOL trial, a pivotal Phase III randomized controlled trial, is currently evaluating the efficacy and safety of MIRV in patients with FRα‐positive platinum‐resistant ovarian cancer. The results suggest significant improvements in the objective response rate (ORR) and progression‐free survival (PFS) compared to standard chemotherapy options, highlighting the potential of FRα‐targeting ADCs as effective second‐line treatments [20].

Given the predominance of single‐arm studies in this field, our study aimed to systematically review and meta‐analyze the existing evidence on the efficacy and safety of FRα‐targeting ADCs, providing a comprehensive and evidence‐based reference for clinicians treating recurrent ovarian cancer.

## 2 Methods

### 2.1 Search Strategy and Eligibility Criteria

This systematic review and meta‐analysis was registered in PROSPERO (CRD42023491151) and performed following the recommendations of the Meta‐analysis of Observational Studies in Epidemiology (MOOSE) and Preferred Reporting Items for Systematic Reviews and Meta‐Analyses (PRISMA) guidelines [21].

Eligible studies were prospective clinical trials that investigated FRα‐targeting ADC therapy in recurrent ovarian cancer. We searched relevant full‐text articles or abstracts from the MEDLINE, Embase, and Cochrane Library databases published up to 7 December 2023. To find relevant articles, the following combination of search words was included: (“FRα” or “FolRα” or “FRalpha” or “folate receptor alpha” or “folate receptor‐alpha” or “FR” or “folate receptor”) and (“ovarian cancer” or “ovarian carcinoma” or “ovarian neoplasm”) (Table S1). For the study purpose, we conducted a comprehensive manual search of conference materials, including abstracts, posters, and presentations, from the websites of the American Society of Clinical Oncology and the European Society of Medical Oncology to ensure a thorough review.

Based on the Patient, Intervention, Comparison, and Outcome approach [22], studies were searched and screened according to the inclusion criteria. We identified studies that met the following conditions: (1) diagnosed with 2014 International Federation of Gynecology and Obstetrics (FIGO) stage III or IV epithelial ovarian, fallopian, or primary peritoneal cancer; (2) underwent second‐line or above treatment of recurrent ovarian cancer; and (3) received ADC targeting FRα after recurrence. Meanwhile, studies were excluded if they were (1) non‐human studies, reviews, meta‐analyses, or case reports, or (2) did not include related data on efficacy and toxicity.

**FIGURE 1:**
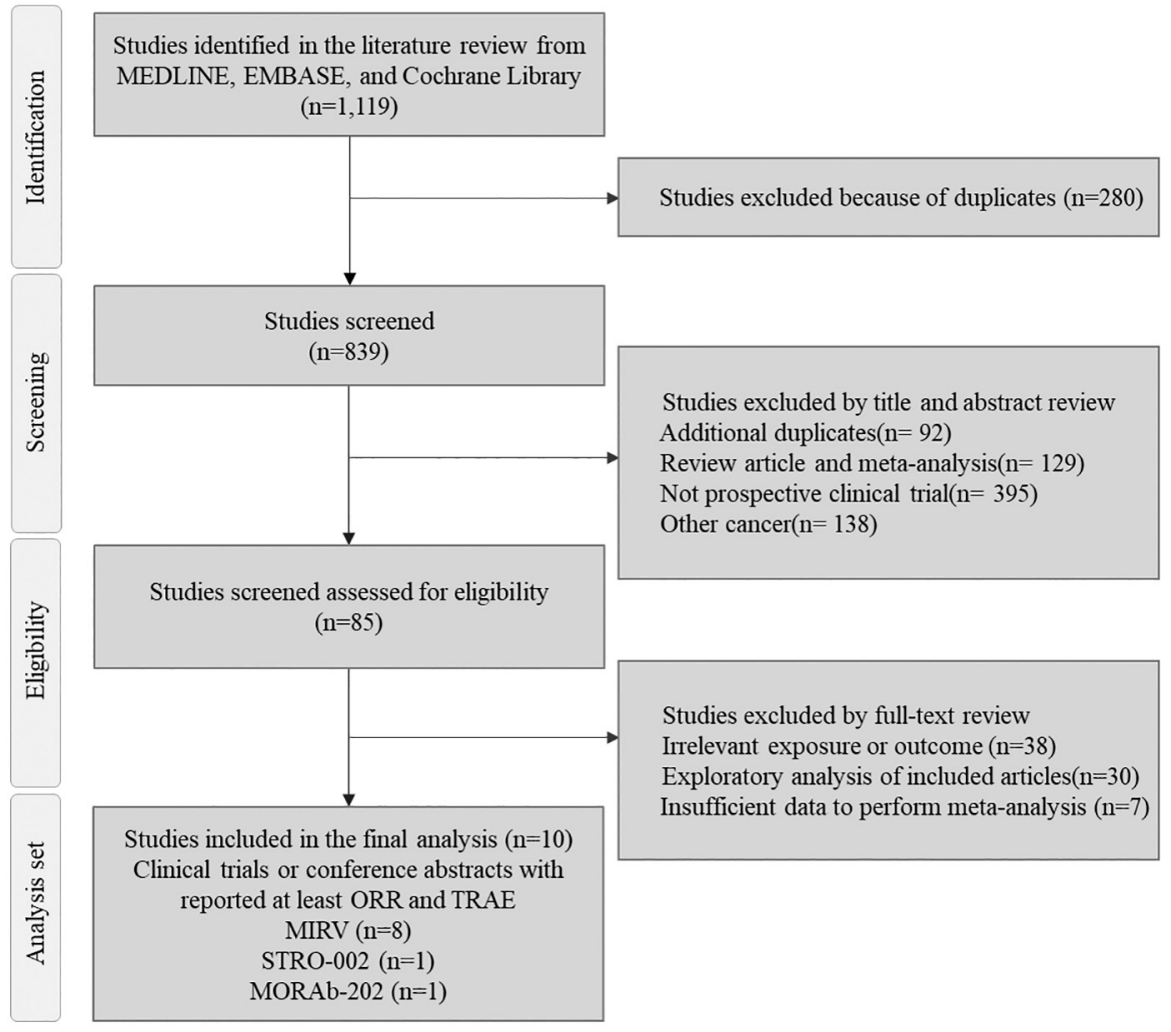
PRISMA flow diagram.

We identified 1119 studies and excluded 280 duplicates. We further excluded studies for the following reasons: additional duplicates (*n* = 92), review articles or meta‐analyses (*n* = 129), non‐prospective clinical trials (*n* = 395), and studies on other cancers (*n* = 138). During the assessment of full‐text articles, we excluded those with irrelevant exposures or outcomes (*n* = 38), exploratory analyses (*n* = 30), and insufficient data to perform meta‐analysis (*n* = 7). Finally, eight clinical trials related to MIRV [17, 18, 19, 20, 23, 24, 25, 26], one clinical trial regarding STRO‐002 [27], and one clinical trial regarding MORAb‐202 [28] were included in this meta‐analysis (Figure 1).

### 2.2 Data Analysis

Two investigators (ETK and JHK) independently reviewed all the extracted literature, and any discrepancies were resolved through consensus after discussion with a third author (MCL). The following data were extracted from each study: author names, clinical trial registration number, year of publication, study design, interventions, median age, median follow‐up period, number of enrolled patients, disease status, FRα expression, treatment‐related adverse events (TRAEs), and ORR (Table 1).

**TABLE 1:** Summary of study characteristics.

Author	Clinical trial registration number	Published year	Study design	Drug	Median age, year (range)	Median follow up, month	Patient numbers	Disease status	FRalpha expression number (%)	Evaluated safety outcome (AEs)
Martin et al. [24]	NCT01609556	2017	Single‐arm phase I	Mirvetuximab Soravtansine, 6 mg/kg; Q3W	62 (38–76)	NA	27	Recurrent EOC	Low 6 (22) Medium 5 (19) **High 16 (59)**	All, Grade ≥ 3, Discontinuation
Moore et al. [17]	NCT01609556	2017	Single‐arm phase I	Mirvetuximab Soravtansine, 6 mg/kg; Q3W	62.5 (41–81)	5.5	46	PROC	Low 9 (19.5) Medium 14 (30.5) **High 23 (50)**	Grade ≥ 3, Discontinuation
O'Malley et al. [26]	NCT02606305	2020	Single‐arm phase I	Mirvetuximab Soravtansine, 6 mg/kg; Q3W **bevacizumab 15 mg/kg, Q3W**	63 (39–81)	14.8	66	PROC	Low 13 (19.7) Medium 24 (36.4) **High 28 (42.4)**NA 1 (1.5)	All, Serious, Discontinuation
Matulonis et al. [18]	NCT04296890	2023	Single‐arm phase II	Mirvetuximab Soravtansine, 6 mg/kg; Q3W	62 (35–85)	13.4	106	PROC	**High 106 (100)**	All, Grade ≥ 3, Serious, Discontinuation
Gilbert et al.—1 [23]	NCT02606305	2023	Single‐arm phase1	Mirvetuximab Soravtansine, 6 mg/kg; Q3W **bevacizumab 15 mg/kg, Q3W**	62 (39–81)	8.7	94	PROC	Low 11 (11.7) Medium 39 (41.5) **High 44 (46.8)**	All, Serious, Discontinuation
Gilbert et al.—2 [23]	NCT02606305	2023	Single‐arm phase1	Mirvetuximab Soravtansine, 6 mg/kg; Q3W **bevacizumab 15 mg/kg, Q3W**	59 (44–83)	8.7	31	PSOC	Low 1 (3) Medium 32 (39) **High 44 (58)**	NA
Moore et al. [19]	NCT02631876	2021	RCT	Mirvetuximab Soravtansine, 6 mg/kg; Q3W	64 (34–89)	12.5	248	PROC	Medium101 (40.7) **High 147 (59.3)**	All, Grade ≥ 3, Serious, Discontinuation
Moore et al. [20]	NCT04209855	2023	RCT	Mirvetuximab Soravtansine, 6 mg/kg; Q3W	64 (32–88)	2.96	227	PROC	**High 227 (100)**	All, Grade ≥ 3, Serious, Discontinuation
Moore et al. [25]	NCT02606305	2018	Single‐arm phase1	Mirvetuximab Soravtansine 5 to 6 mg/kg;Q3W **Carboplatin AUC4 to 5 Q3W**	66 (47–82)	15.9	18	PSOC	Low 7 (38.9) Medium 4 (22.2) **High 7 (38.9)**	Serious, Discontinuation
Nishio et al.^a^ [28]	NCT03382942	2022	Double –arm Phase 1	MORAb‐202 0.9 mg/kg; Q3W	NA	NA	24	PROC	NA	Grade ≥ 3
				MORAb‐202 1.2 mg/kg; Q3W	NA	NA	21	PROC	NA	
Oaknin et al.^a^ [27]	NCT03748186	2023	Double –arm Phase 1	STRO‐002 4.3 mg/kg; Q3W	NA	NA	16	Recurrent EOC	FRα TPS > 25%	NA
				STRO‐002 5.2 mg/kg; Q3W	NA	NA	16	Recurrent EOC	FRα TPS > 25%	

*Note:* The bold values in the "Drug" row indicate the significance for combination treatment drugs. In the "FRalpha expression number (%)" row, the bold values indicate the rate of high FRalpha expression. This meta‐analysis was conducted with subgroup analyses of monotherapy vs. combination therapy and high FRalpha expression, and bold values were used for reader convenience.

Abbreviations: AE, adverse effect; AUC, area under the curve; EOC, epithelial ovarian cancer; NA, not available; PROC, platinum‐resistant ovarian cancer; PSOC, platinum sensitive ovarian cancer; Q3W, once every 3 weeks; TPS, tumor proportion score.

^a^ Refer to study that reported abstract and presented congress of ASCO 2022, 2023 respectively.

The primary endpoint was the ORR based on RECIST 1.1 criteria. We conducted a further analysis of TRAEs and ORR in subgroups based on FRα expression and disease status.

To assess the methodological quality of the one‐arm study, a methodological non‐randomized study (MINORS) indicator was used. Quality assessment of the included studies was performed using the Risk of Bias‐2 (RoB‐2) tool, as two of the screened studies were randomized controlled trials (Table S2).

To evaluate the heterogeneity among the studies, we employed Higgins' *I* ^2^ statistics [29]. If *I* ^2^ was less than 50%, a fixed‐effect model was employed to determine the pooled proportion and its 95% confidence interval (CI). Conversely, if *I* ^2^ exceeded 50% or the p‐value was less than 0.05, we investigated the sources of heterogeneity and performed a subgroup analysis to identify contributing factors. Statistical analyses were performed using R software version 4.1.1 (R Foundation for Statistical Computing, Vienna, Austria).

## 3 Results

### 3.1 Clinical Characteristics

A total of 940 patients from 10 studies with 13 arms was included in this meta‐analysis. All studies were performed in a recurrent setting: platinum‐sensitive relapse in one single‐arm study and platinum‐resistant relapse in seven studies, including two RCTs. Among these studies related to MIRV, five used MIRV monotherapy, two used MIRV in combination with bevacizumab, and one in combination with carboplatin. The study by Nishio *et al*. investigated MORAb‐202 at two dosage levels. The study by Oaknin *et al*. evaluated STRO‐002 at two dosage levels and included patients with high FRα expression (TPS > 25%). All 10 studies provided ORR data, and six studies provided subgroup cohorts of ORR in patients with high FRα expression. Moreover, six studies provided the incidence of adverse events (AEs) and seven studies, including MoRAb‐202, provided the incidence of ≥ grade 3 AEs. Serious AEs and AEs leading to drug discontinuation were reported in seven studies (Table 1).

### 3.2 Efficacy

**FIGURE 2:**
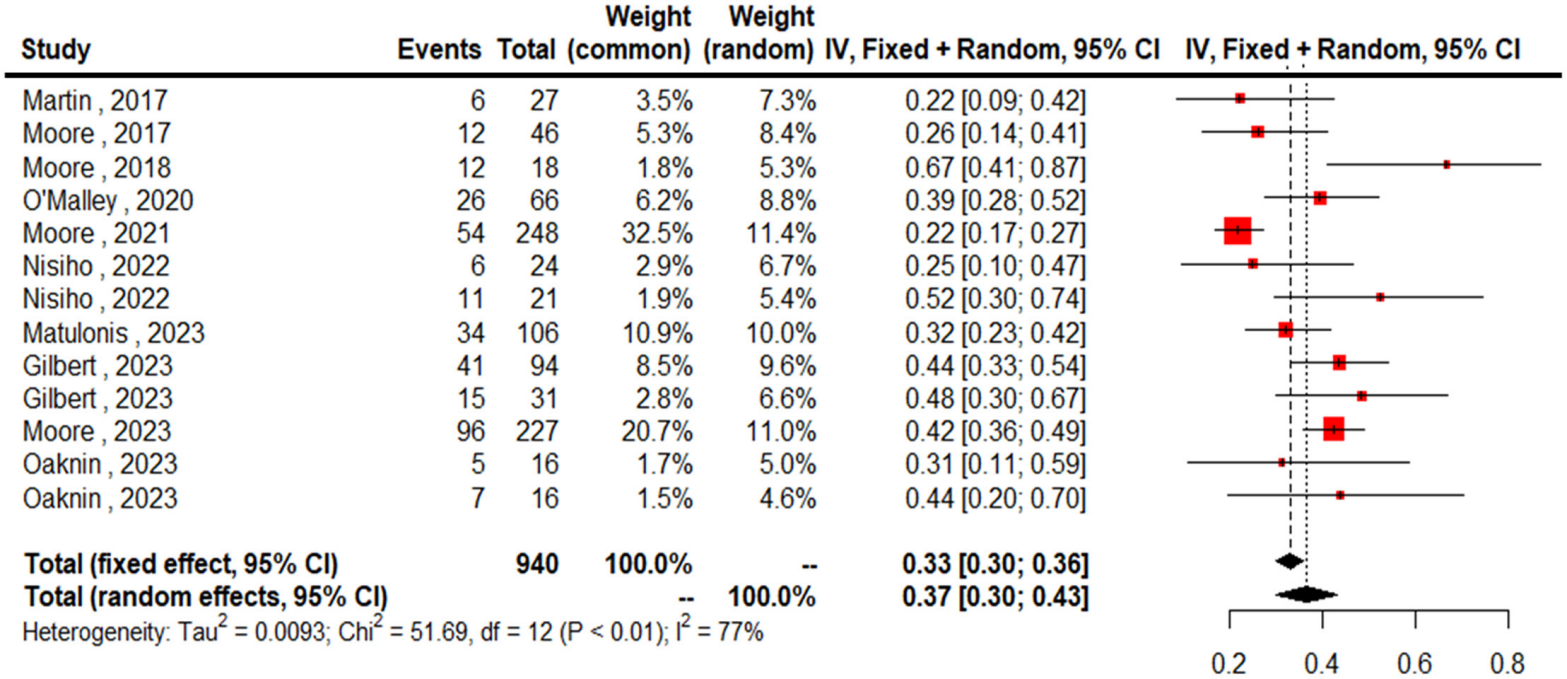
Forest plots of pooled ORR.

**FIGURE 3:**
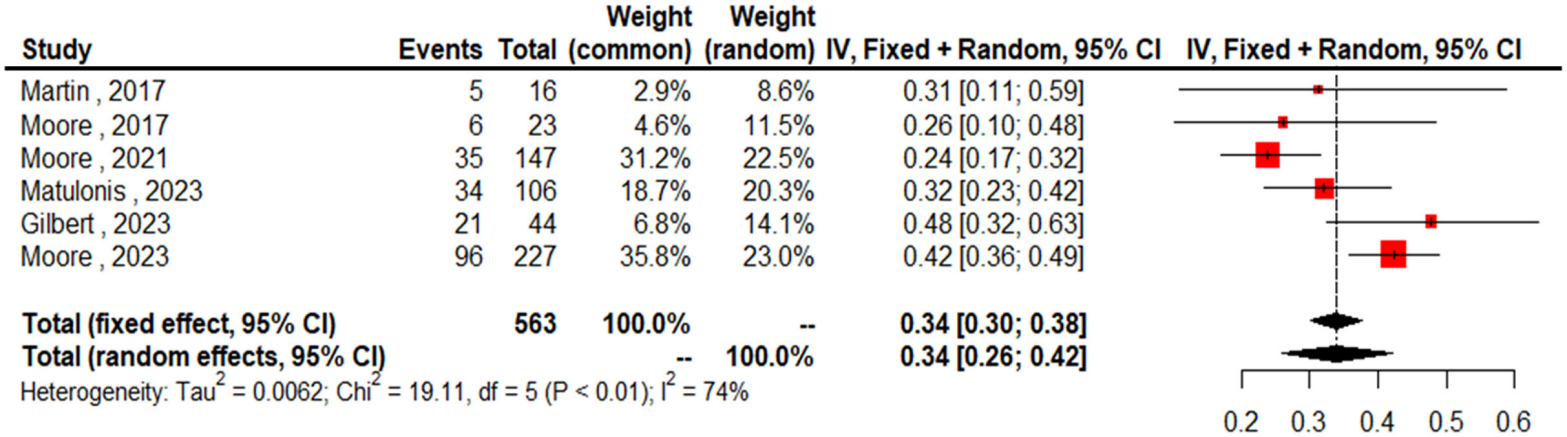
Forest plots of pooled ORR in the high‐folate receptor alpha expression population.

The combined ORR using the random‐effect model was 0.37 (95% CI: 0.30–0.43; Figure 2). The heterogeneity among the studies was substantial (*I* ^2^ = 77%), indicating significant variability in the treatment effects across the included studies. Figure 3 presents the forest plot of the ORR according to FRα expression rate, specifically in the high expression subgroup. The combined ORR for the high expression group was 0.34 (95% CI: 0.26–0.42). In the subgroup analysis according to disease status, the ORR for platinum‐resistant ovarian cancer (PROC) patients was 0.35 (95% CI: 0.27–0.42, *I* ^2^ = 81%). In the platinum‐sensitive ovarian cancer (PSOC) group, the ORR was 0.56 (95% CI: 0.39–0.74, *I* ^2^ = 39%; Figure S1). These findings show variability in treatment efficacy based on disease status, with the highest ORR observed in the PSOC group. A detailed efficacy profile related to the studies in this meta‐analysis is provided (Table 2). ADCs not targeting FRα are being studied for gynecologic malignancies, each targeting different antigens. Table 3 summarizes non‐FRα ADCs, highlighting their target antigens, mechanisms, and advancements in clinical trials with patients with ovarian cancer.

**TABLE 2:** Summary of study efficacy profile.

Author	ORR number (%) [95% CI]	CR number (%)	PR number (%)	Median DOR months [95% CI]	Median PFS months [95% CI]	Median OS months [95% CI]	ORR with high FRα expression number (%) [95% CI]	Median PFS with high FRα expression months [95% CI]	Median OS with high FRα expression months [95% CI]
Martin et al. [24]	6 (22.2) [6.5–37.9]	2 (7.4)	4 (14.8)	NA	4.2 [2.8–5.4]	NA	5 (31.3) [8.5–54.0]	5.4 [2.8—NR]	NA
Moore et al. [17]	12 (26) [14.3–41.1]	1 (2)	11 (24)	19.1 [16.1–33.1]	4.8 [3.9–5.7]	NA	6 (26.1) [10.2–48.4]	NA	NA
O'Malley et al. [26]	26 (39) [28–52]	5 (7.5)	21 (31.5)	8.6 [4.9–14.9]	6.9 [4.9–8.6]	NA	NA	NA	NA
Matulonis et al. [18]	34 (32.4) [23.6–42.2]	5 (4.8)	29 (27.6)	6.9 [5.6–9.7]	5.5 [3.8–6.9]	13.8 [12.0—NR]	34 (32.4) [23.6–42.2]	5.5 [3.8–6.9]	13.8 [12.0—NR]
Gilbert et al.—1 [23]	41 (44) [33–54]	5 (5)	36 (38)	9.7 [6.9–14.1]	8.2 [6.2–10.0]	NA	21 (48) [33–63]	9.7 [6.8–11.0]	NA
Gilbert et al.—2 [23]	15 (48) [30–67]	12 (39)	14 (45)	12.7 [5.0–14.5]	9.6 [5.4–14.1]	NA	NA	NA	NA
Moore et al. [19]	54 (22) [16.6–26.9]	NA	NA	5.7 [NA]	4.1 [0.73–1.31]	16.4 [0.58–1.15]	35 (24) [16.9–30.7]	4.8 [0.48–1.00]	NR [0.40–0.97]
Moore et al. [20]	96 (42.3) [35.8–49]	12 (5.3)	84 (37.0)	6.77 [5.62–8.31]	5.62 [4.34–5.95]	16.46 [14.46–24.57]	96 (42.3) [35.8–49]	5.62 [4.34–5.95]	16.46 [14.46–24.57]
Moore et al. [25]	12 (71) [44–90]	3 (17.75)	9 (53.25)	NA	15 [9.9–NR]	NA	NA	NA	NA
Nishio et al.^a^ [28] MORAb‐202 0.9 mg/kg; Q3W	6 (25.0) [9.8–46.7]	1 (4.2)	5 (20.8)	NA	6.7 [1.5–12.0]	10.5 [6.4–15.1]	NA	NA	NA
Nishio et al.^a^ [28] MORAb‐202 1.2 mg/kg; Q3W	11 (52.4) [29.8–74.3]	0 (0)	11 (52.4)	NA	8.2 [4.2–10.4]	NA [12.5–NR]	NA	NA	NA
Oaknin et al.^a^ [27] STRO‐002 4.3 mg/kg; Q3W	5 (31.3) [11.0–58.7]	NA	NA	13 [4.5–NR]	6.1 [4.0–8.3]	NA	NA	NA	NA
Oaknin et al.^a^ [27] STRO‐002 5.2 mg/kg; Q3W	7 (43.8) [19.8–70.1]	NA	NA	5.4 [2.4–6.1]	6.6 [2.9–7.6]	NA	NA	NA	NA

Abbreviations: CI, confidence interval; CR, complete response; DOR, duration of response; FRα, folate receptor alpha; NA, not available; NR, not reached; ORR, objective response rate; OS, overall survival; PFS, progression‐free survival; PR, partial response.

^a^ Refer to study that reported abstract and presented congress of ASCO 2022, 2023 respectively.

**TABLE 3:** Non‐FRα targeted antibody‐drug conjugates under clinical investigation in ovarian cancers.

Target antigen	ADC	Cytotoxic payload	Linker type	Clinical trial registration number	Study design	Disease status	Key findings
Mesothelin	Anetumab Ravtansine	DM4	Non‐cleavable disulfide‐containing linker	NCT03587311	Phase II	Platinum‐resistant ovarian cancer	ORR ~18%, median PFS ~5.3 months; common AEs include increased AST (71%), ALT (64%), thrombocytopenia (61%), and peripheral neuropathy (46%); study terminated due to better outcomes with control arm
NaPi2b	Upifitamab Rilsodotin (UpRi)	AF‐HPA	Protease cleavable thioether bond	NCT03319628	Phase II	Platinum‐resistant ovarian cancer	Primary endpoint (investigator‐assessed ORR) not met. ORR ~15.6% in NaPi2b‐positive patients, median DOR 7.4 months; overall ORR ~13.1% with similar DOR (7.4 months)
Trop‐2	Sacituzumab Govitecan	SN‐38	Cleavable CL2A linker	NCT04251416	Phase II	Recurrent epithelial ovarian cancer	Preliminary efficacy in advanced relapsed endometrial cancer with ORR ~22%. Ongoing phase II study in epithelial ovarian cancer (NCT04152499)
HER2	Trastuzumab Deruxtecan	Deruxtecan (DXd)	Tetrapeptide‐based cleavable linker	NCT04482309	Phase II	HER2‐expressing epithelial ovarian cancer	ORR 45%, median PFS 5.9 months (IHC 3+ at 12.5 months), median OS 13.2 months (IHC 3+ at 20.0 months)
B7‐H4	SGN‐B7H4V	MMAE	Cleavable maleimidocaproyl valine‐citrulline (mc‐vc) linker	NCT05194072	Phase I	Epithelial ovarian cancer with prior platinum therapy	Preliminary ORR ~13% in ovarian cancer cohort. Manageable safety profile observed, with dose‐limiting toxicities included fatigue (~27.5%) and peripheral neuropathy (~27.5%)

Abbreviations: ALT, alanine aminotransferase; AST, aspartate aminotransferase; DOR, duration of response; IHC, immunohistochemistry; ORR, objective response rate; PFS, progression‐free survival.

### 3.3 Safety

**FIGURE 4:**
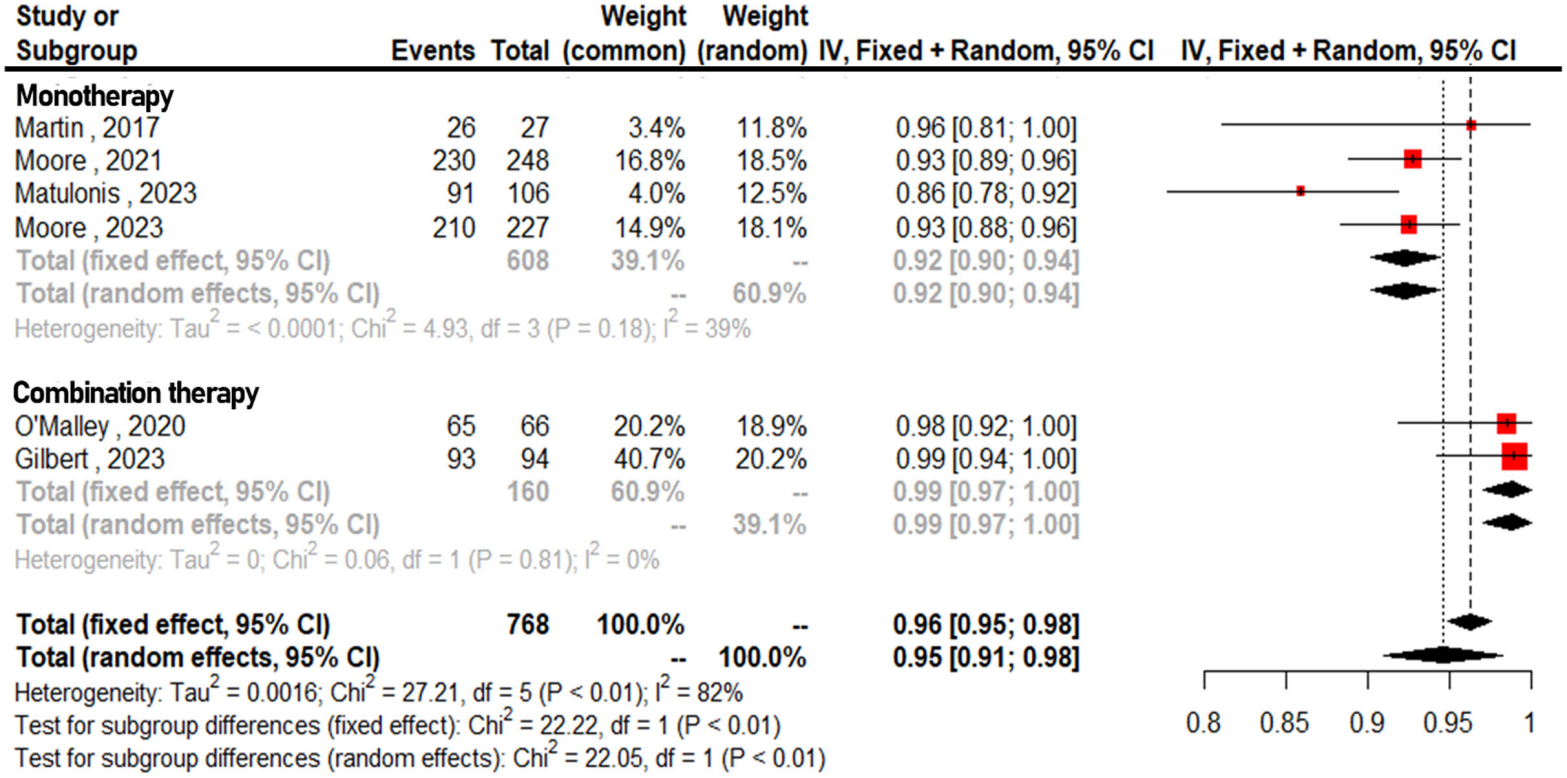
Forest plots for pooled incidence of overall treatment‐related adverse events.

**FIGURE 5:**
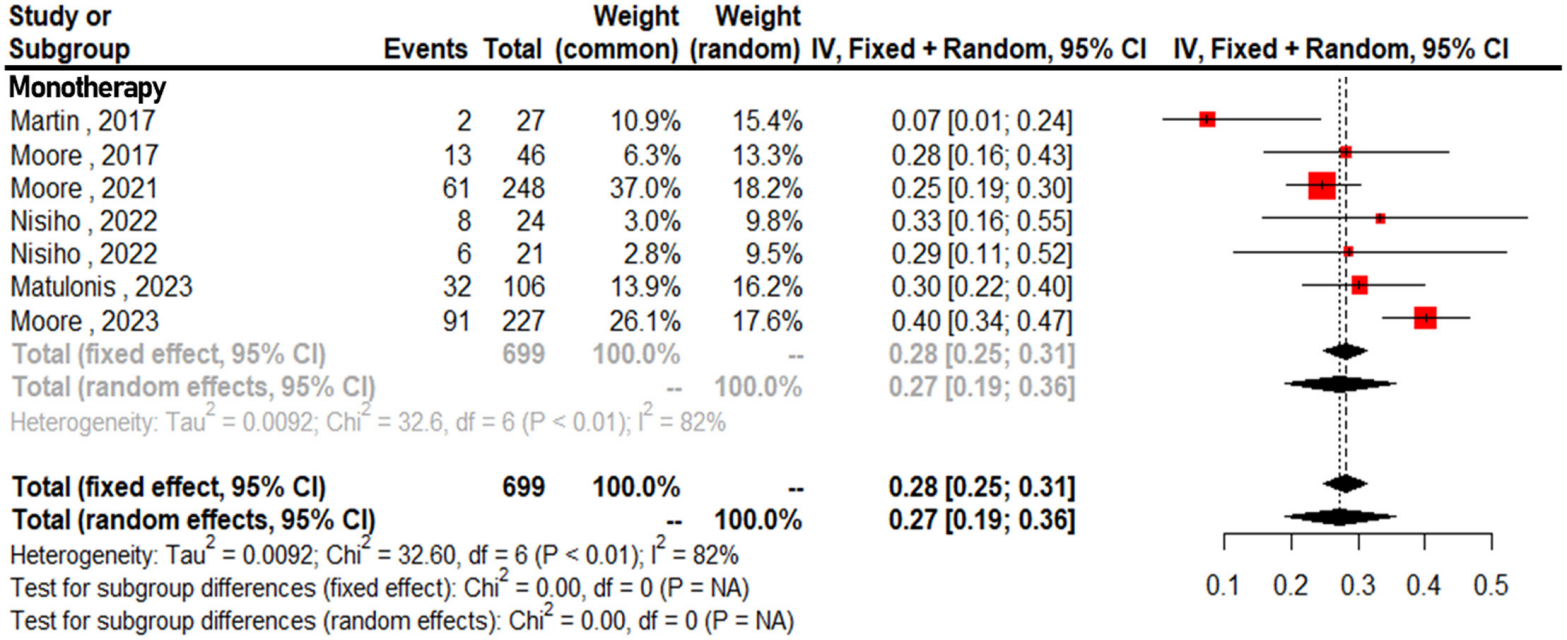
Forest plots for pooled incidence of overall treatment‐related ≥ grade 3 adverse events.

Figure 4 displays the forest plots for the pooled incidence of overall TRAEs, which was 0.95 across all studies (95% CI: 0.91–0.98, *I* ^2^ = 82%). The analysis distinguishes between monotherapy and combination therapy. The pooled incidence of TRAEs for monotherapy was 0.92 (95% CI: 0.90–0.94, *I* ^2^ = 39%), while that for combination therapy was 0.99 (95% CI: 0.97–1.00, *I* ^2^ = 19%). Analysis of subgroups showed a statistically significant difference in TRAEs (*p* < 0.01). The combined incidence of grade ≥ 3 adverse events was 0.27 (95% CI: 0.19–0.36, *I* ^2^ = 82%). All studies in this analysis involved monotherapy (Figure 5). The overall incidence of serious AEs was 0.21 (95% CI: 0.10–0.32). When analyzed by treatment regimen, the incidence of monotherapy was 0.15 (95% CI: 0.07–0.24, *I* ^2^ = 90%), while that for combination therapy was 0.29 (95% CI: 0.06–0.52, *I* ^2^ = 41%; Figure S2). Subgroup analysis showed no significant difference (*p* = 0.28). The combined incidence of AEs leading to drug discontinuation was 0.12 (95% CI: 0.03–0.21). For monotherapy, the incidence was 0.07 (95% CI: 0.04–0.09, *I* ^2^ = 46%), while that for combination therapy was 0.20 (95% CI: 0.10–0.30, *I* ^2^ = 94%). Subgroup analysis showed no significant difference (*p* = 0.20; Figure S3).

### 3.4 Publication Bias Analysis

To assess publication bias, we used funnel plots with effect size on the x‐axis and standard error of the log HR on the y‐axis. The funnel plot appears relatively symmetrical, further supporting the lack of significant publication bias in the meta‐analysis. The results of Egger's test (*p* = 0.141) and Begg's test (*p* = 0.542) also supported the absence of publication bias (Figure S4).

## 4 Discussion

The current analysis demonstrated that FRα‐targeted ADCs exhibited a promising ORR and PFS, with the rate of TRAEs providing an additional reference value. The pooled ORR was 0.37 (95% CI: 0.30–0.43). Although not all included studies provided relevant data, the study cohort with high expression of FRα receptors had a similar response (ORR 0.34, 95% CI: 0.26–0.42) to FRα‐targeted ADCs compared to the overall group.

Based on previous studies, patients exhibiting elevated levels of FRα receptors demonstrated a comparatively greater response to FRα‐targeted ADCs [18, 30]. The lessons learned from the results of the FORWARD I study revealed that the maximal benefit of MIRV was observed in patients with high FRα expression [19]. A detailed analysis of the study data revealed that using membranous staining visible at a 10x microscope objective (referred to as 10x scoring) as the method for determining FRα positivity in enrolled patients may have been insufficiently accurate in assessing actual FRα expression levels. Exploratory analyses that re‐evaluated the data using PS2+ scoring indicated that the 10x scoring method likely allowed the inclusion of patients with lower‐than‐anticipated FRα expression levels [31]. This misclassification likely diminished the observed effectiveness of MIRV, underscoring the critical role of accurately identifying high FRα expression to achieve the maximal therapeutic benefit. Another perspective is that recent studies on ADCs have demonstrated a high ORR across various cancers, irrespective of the target receptor expression levels [32, 33]. Although there were conflicting results on the impact of expression levels on efficacy, our study demonstrates similar ORRs between the overall cohort and the high‐FRα expression cohort. This result may be attributed to the bystander effect, a unique mechanism wherein the cytotoxic payload of an ADC, once released inside the target cancer cell, diffuses into neighboring cells, exerting its lethal effect on both antigen‐positive and antigen‐negative cells within the tumor microenvironment [34, 35]. Trastuzumab deruxtecan (T‐DXd), a humanized anti‐HER2 IgG1 antibody [36], has shown effectiveness not only in HER2‐overexpressing tumors but also in HER2‐low tumors [33]. The effectiveness of T‐DXd in achieving a high ORR in low‐HER2‐expressing tumors illustrates the potential adaptability and efficacy of ADCs across various levels of receptor expression. Future research should focus on the expression levels responsive to FRα‐targeted ADCs. Our analysis of AEs revealed that nearly all patients experienced at least one reaction. The overall pooled incidence of TRAEs was 0.95 (95% CI: 0.91–0.98). However, the occurrence of grade 3 or higher AEs was significantly reduced to 0.27 (95% CI: 0.19–0.36). We categorized the included studies of the overall pooled incidence of TRAEs into two subgroups based on therapy regimen of monotherapy or combination therapy. The test for subgroup differences of the overall pooled incidence of TRAEs showed a significant difference (*p* < 0.01), indicating that the incidence of TRAEs varied between the monotherapy and combination therapy groups. However, the incidence of serious AEs was similar in the combination therapy group compared to the MIRV monotherapy group (15% vs. 29%, *p* = 0.28). Additionally, the pooled discontinuation rate due to AEs for the two subgroups was 7% vs. 20% (*p* = 0.20). These findings suggest that FRα‐targeted ADCs have an acceptable safety profile for patients with EOC.

In ovarian cancer, FRα is overexpressed in approximately 80% of cases of EOCs [37]. Unlike mesothelin and CA125, which have a broader expression in normal tissues, the differential expression of FRα in tumors compared to normal tissues enhances tumor selectivity for drugs [38]. By actively internalizing through receptor‐mediated endocytosis, it promotes the administration of ADC directly into tumor cells [39, 40]. Notably, FRα expression persists in metastatic and recurrent tumors and remains unaffected by prior chemotherapy, making it a reliable target throughout the course of the disease [41]. High FRα expression is also linked to chemotherapy resistance, further underscoring its potential as a therapeutic target in solid tumors [42]. Given its minimal expression in normal tissues, its involvement in cancer progression, and its strong affinity for non‐natural ligands like folic acid, FRα has emerged as an ideal therapeutic target. It is suitable for various drugs, such as monoclonal antibodies, small‐molecule inhibitors, and antibody‐drug conjugates, providing multiple avenues for targeted cancer therapy [34].

Two notable agents from earlier generations, vintafolide and farletuzumab, demonstrated both the potential and challenges of targeting FRα in cancer therapy. Vintafolide, a folate conjugate of desacetylvinblastinemonohydrazide, uses the FRα‐mediated endocytosis pathway to deliver its cytotoxic payload into tumor cells [39]. Early‐phase clinical trials, including phase I and multiple phase II studies, showed promising results in various solid tumors, such as lung and ovarian cancer [43, 44]. The phase II PRECEDENT study (NCT00722592) showed significant prolongation of PFS (hazard ratio [HR], 0.63; 95% CI, 0.41 to 0.96; *p* = 0.031) in patients with platinum‐resistant ovarian cancer when combined with PEGylated liposomal doxorubicin (PLD) [45]. However, the phase III PROCEED trial (NCT01170650) was discontinued during the interim analysis due to failure to meet the pre‐specified primary outcome for PFS improvement [39, 46]. Farletuzumab (MORab003) is a fully humanized IgG1 monoclonal antibody specific for FRα [39]. Unlike other agents, it does not interfere with folate binding or transport but targets FRα‐expressing tumor cells through mechanisms like antibody‐dependent cellular cytotoxicity and complement‐dependent cytotoxicity [13]. A phase II trial (NCT00318370) demonstrated that farletuzumab combined with carboplatin and a taxane, followed by maintenance therapy, had favorable response rates and was well‐tolerated without increasing toxicity [47]. Building on these promising results, a large phase III trial (NCT00849667) was conducted to evaluate the same combination in the same patient population. Unfortunately, this trial did not achieve its primary endpoint of improved progression‐free survival (PFS), although further analysis indicated improved PFS in subgroups with higher doses and lower CA125 levels [48]. Subsequently, a phase II study was initiated to confirm the survival gain in these specific subgroups but failed to reach the PFS endpoint [49]. The limited efficacy observed with these earlier FRα‐targeting agents may be attributed to several factors. First, the diverse patient populations in these trials resulted in heterogeneous expression of FRα both in ovarian cancer and within the study cohorts. This variability likely influenced the overall therapeutic outcomes of agents like farletuzumab and vintafolide. Studies have shown that FRα expression can vary significantly among patients, leading to inconsistent responses to FRα‐targeted therapies [13, 49, 50]. Second, suboptimal drug delivery mechanisms may have played a critical role. The mechanisms employed by these agents seemed to not efficiently deliver the cytotoxic payload directly into the cancer cells, leading to insufficient therapeutic concentrations at the tumor site. Improving the specificity and efficiency of drug delivery remains a crucial area for future research to enhance the effectiveness of FRα‐targeted therapies.

**FIGURE 6:**
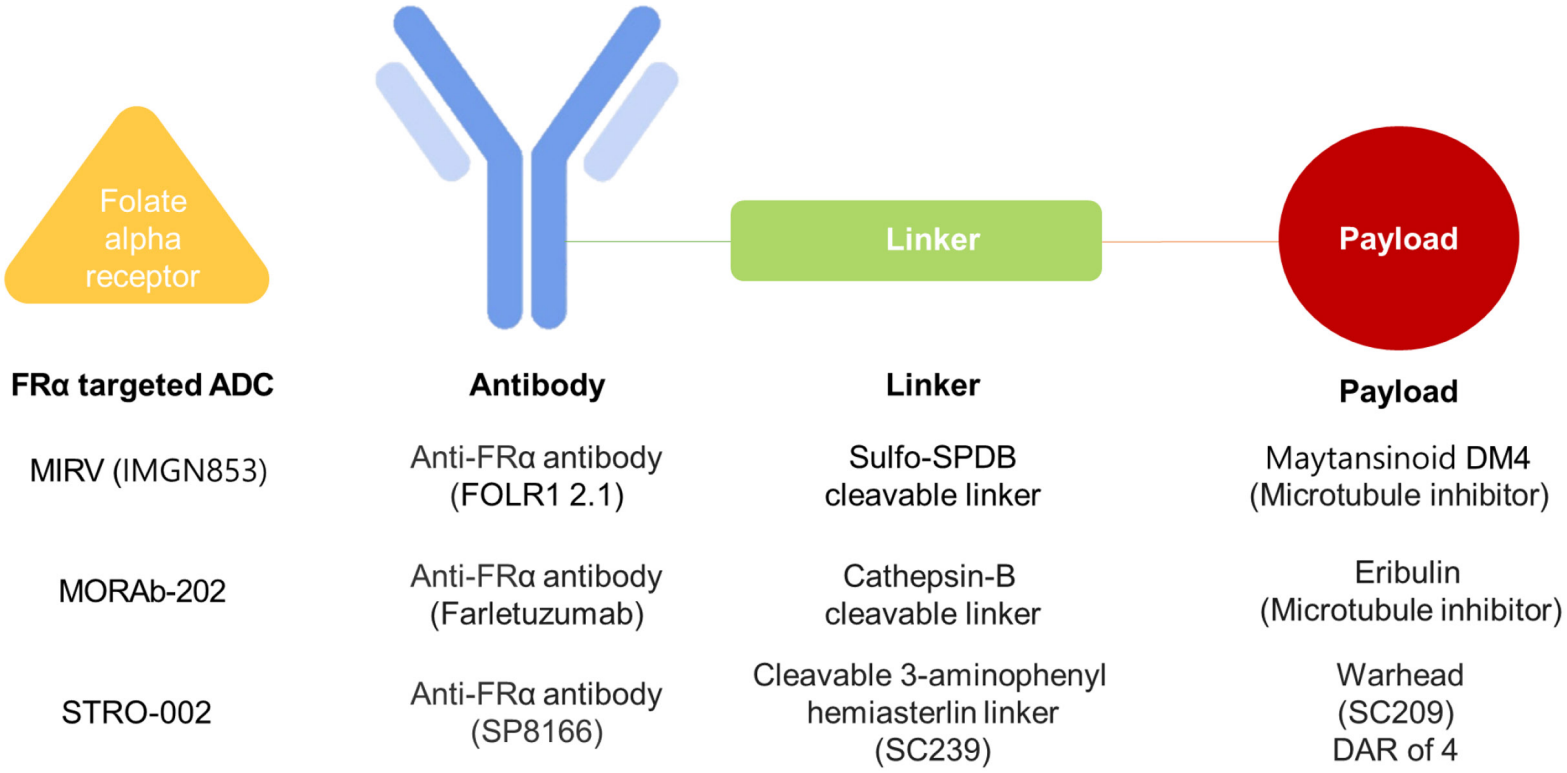
Structural components of FRα‐targeted antibody‐drug conjugates.

Recent advancements in ADC technology have focused on improving targeting and drug delivery mechanisms. Enhanced targeting involves optimizing the antibody, linker, and payload components to increase specificity and efficacy [12]. The bystander effect is a significant mechanism by which the cytotoxic payload can diffuse into neighboring cancer cells, affecting both antigen‐positive and antigen‐negative cells within the tumor microenvironment [51, 52]. To date, several ADCs targeting the FRα receptor have been successful in clinical trials. MORAb‐202, STRO‐002, and MIRV are three FRα‐targeted ADCs that utilize distinct cytotoxic agents and linker designs, potentially leading to varied clinical outcomes and toxicity profiles. MORAb‐202 combines farletuzumab with eribulin, a microtubule inhibitor with unique activities, including vascular remodeling and antimitotic effects [53]. Its cathepsin‐B cleavable linker releases the payload specifically in lysosomal compartments, limiting systemic toxicity. STRO‐002 employs 3‐aminophenyl hemiasterlin (SC209), a tubulin‐targeting agent less prone to drug resistance due to low efflux by P‐glycoprotein pumps. The cleavable linker enables stable circulation and significant bystander killing, observed in FolRα‐negative cells cocultured with target‐positive cells [54]. MIRV incorporates DM4, a potent antimitotic agent that disrupts tubulin polymerization. Its Sulfo‐SPDB cleavable linker allows DM4 to diffuse into surrounding tumor cells, adding a bystander effect [17]. These differences indicate that while each ADC aims to deliver cytotoxic effects specifically to tumor cells, their payloads, linker characteristics, and release strategies impact both therapeutic potential and side effect profiles **(**Figure 6 **)**. Notable outcomes were observed in the early clinical studies for MORAb‐202 and STRO‐002, both targeting FRα in ovarian cancer [12]. In a phase I dose escalation study (STRO‐002‐GM1), STRO‐002 was tested on patients with recurrent epithelial ovarian cancer, including those who were platinum‐resistant or platinum‐sensitive. The study showed an ORR of 31.3% (4.3 mg/kg) to 48.3% (5.2 mg/kg), demonstrating activity at both doses. The treatment was effective even in cancers with lower FRα expression levels, with a cutoff greater than 25%. The most common grade 3 TRAEs were neutropenia (70.5%), arthralgia (18.2%), and anemia (13.6%). Grade 4 neutropenia was more frequent at the higher dose (52% vs. 22%) [27]. MORAb‐202 comprises the humanized anti‐human FRα antibody farletuzumab linked to the chemotherapeutic agent eribulin via a cathepsin‐B cleavable linker. A phase Ib dose expansion trial (NCT03386942) included patients who had undergone up to two regimens of chemotherapy for platinum‐resistant or platinum‐sensitive EOC with positive FRα expression. The trial demonstrated significant activity with an ORR ranging from 25% at 0.9 mg/kg to 52% at 1.2 mg/kg [28].

MIRV demonstrated notable clinical efficacy for patients with FRα‐positive platinum‐resistant ovarian cancer. The phase III SORAYA trial established MIRV's ability to achieve a 32% objective response rate and a median PFS of 4.3 months [18], leading to its accelerated FDA approval for this patient population. Building on these results, the phase III MIRASOL trial confirmed MIRV's superiority over standard chemotherapy, with a 42% ORR compared to 16% for chemotherapy and improved median PFS and OS (HR, 0.67; 95% CI, 0.50 to 0.89; *p* = 0.0005) [20]. MIRV is being actively investigated in several clinical trials. The phase II single‐arm PICCOLO trial (NCT05041257) is evaluating MIRV as a monotherapy for patients with FRα‐positive platinum‐sensitive ovarian cancer who have received 1–3 prior therapies. Additionally, the phase III randomized GLORIOSA trial (NCT05445778) is comparing maintenance therapy with MIRV plus bevacizumab versus bevacizumab monotherapy in patients with FRα‐positive, platinum‐sensitive, high‐grade serous epithelial ovarian, fallopian tube, or primary peritoneal cancers. In line with the new GCIG recommendations [55], the MIROVA trial (NCT04274426) includes patients with a platinum‐free interval > 3 months, broadening eligibility to encompass varying degrees of platinum sensitivity and evaluating all histologic subtypes of ovarian carcinoma, including carcinosarcoma and low‐grade ovarian cancer [56]. By specifically targeting patients with FRα‐high epithelial ovarian, fallopian tube, or peritoneal cancers, the trial shifts toward a more targeted treatment approach, focusing on FRα expression rather than solely on platinum sensitivity. This approach enables evaluation of MIRV with carboplatin across different levels of platinum response, reflecting a shift toward individualized treatment [42, 57]. These trials aim to further establish the efficacy and safety of MIRV in different treatment settings and patient populations.

Studies on immune checkpoint inhibitors (ICI)s have not yet demonstrated a clear advantage of immunotherapy over chemotherapy in ovarian cancer [58]. Currently, there are no clinical data available on the combination of ICIs with MIRV specifically in ovarian cancer, leaving a gap in evidence for this approach in FRα‐positive ovarian cancer. However, promising data have emerged from the use of MIRV with pembrolizumab, an ICI, in microsatellite‐stable (MSS) recurrent or persistent endometrial cancer—a similar FRα‐positive gynecologic cancer [59]. This combination may help overcome the limited efficacy of checkpoint inhibitors in MSS/proficient mismatch repair (pMMR) cases. The study included patients with MSS/pMMR endometrial cancer who had FRα‐positive tumors and received 1–3 prior therapies, with prior ICI use permitted. Results demonstrated an ORR of 37.5% among the 16 treated patients, including one complete and five partial responses, and 31.3% of patients achieved stable disease. Additionally, two patients were progression‐free at 6 months. Although this small‐scale study offers preliminary evidence, further data comparing MIRV monotherapy to its combination with ICIs are necessary to validate these findings and determine the full potential of this combination.

This study presents several strengths and limitations in evaluating the efficacy and safety of FRα‐targeting ADCs in recurrent ovarian cancer. A major strength is the comprehensive analysis of multiple studies, encompassing a diverse patient population, which enhances the generalizability of the findings. The use of robust statistical methods to assess outcomes such as the ORR and safety profiles further supports the reliability of the conclusions. However, this study has several limitations. The reliance on single‐arm trials for much of the data introduces potential biases and limits the strength of evidence. The heterogeneity among included studies, in terms of patient populations and treatment protocols, may also affect the comparability of results. Moreover, the relatively recent clinical application of these drugs means that long‐term efficacy and safety data are lacking. Future research should aim to include more randomized controlled trials to provide higher‐quality evidence and investigate the long‐term outcomes of FRα‐targeting ADCs.

## 5 Conclusion

In conclusion, our meta‐analysis highlights the potential of FRα‐targeting ADCs in the treatment of recurrent ovarian cancer. The analysis reveals clinical benefits regardless of FRα expression level in patients. Despite the inherent limitations of single‐arm trials and heterogeneity among included studies, the consistent efficacy and manageable safety profile of these therapies provide a compelling case for their continued development and clinical application. The accelerated approval of MIRV and the encouraging results from early studies of STRO‐002 and MORAb‐202 further support the potential of FRα‐targeted treatments.

## Author Contributions

**Eun Taeg Kim:** conceptualization (equal), data curation (equal), formal analysis (equal), funding acquisition (equal), investigation (equal), methodology (equal), project administration (equal), writing – original draft (equal), writing – review and editing (equal). **Ji Hyun Kim:** conceptualization (equal), data curation (equal), formal analysis (equal), investigation (equal), methodology (equal), writing – original draft (equal), writing – review and editing (equal). **Eun Young Park:** formal analysis (equal), methodology (equal), software (equal), validation (equal), visualization (equal). **In Hye Song:** data curation (equal), formal analysis (equal), investigation (equal), writing – original draft (equal). **Han Song Park:** data curation (equal), formal analysis (equal), methodology (equal), writing – original draft (equal). **Sang‐Yoon Park:** conceptualization (equal), resources (equal), supervision (equal), validation (equal), visualization (equal), writing – review and editing (equal). **Myong Cheol Lim:** conceptualization (equal), formal analysis (equal), funding acquisition (equal), investigation (equal), methodology (equal), project administration (equal), supervision (equal), writing – original draft (equal), writing – review and editing (equal).

## Ethics Statement

This study did not involve human participants and was approved by the Institutional Review Board of the National Cancer Center, Korea. (NCC2024‐0208).

## Conflicts of Interest

The authors declare no conflicts of interest.

## Supporting information


Figure S1.

Figure S2.

Figure S3.

Figure S4.

Table S1.
**Table S2**.

## Data Availability

The data that support the findings of this study are available from the corresponding author upon reasonable request.
