# Supplementary figures and images for: The Efficacy and Safety of Folate Receptor α‐Targeted Antibody‐Drug Conjugate Therapy in Patients With High‐Grade Epithelial Ovarian, Primary Peritoneal, or Fallopian Tube Cancers: A Systematic Review and Meta‐Analysis
Source: Cancer Med. 2024 Nov 11;13(21):e70392. doi: 10.1002/cam4.70392 (PMC11551784; doi:10.1002/cam4.70392)

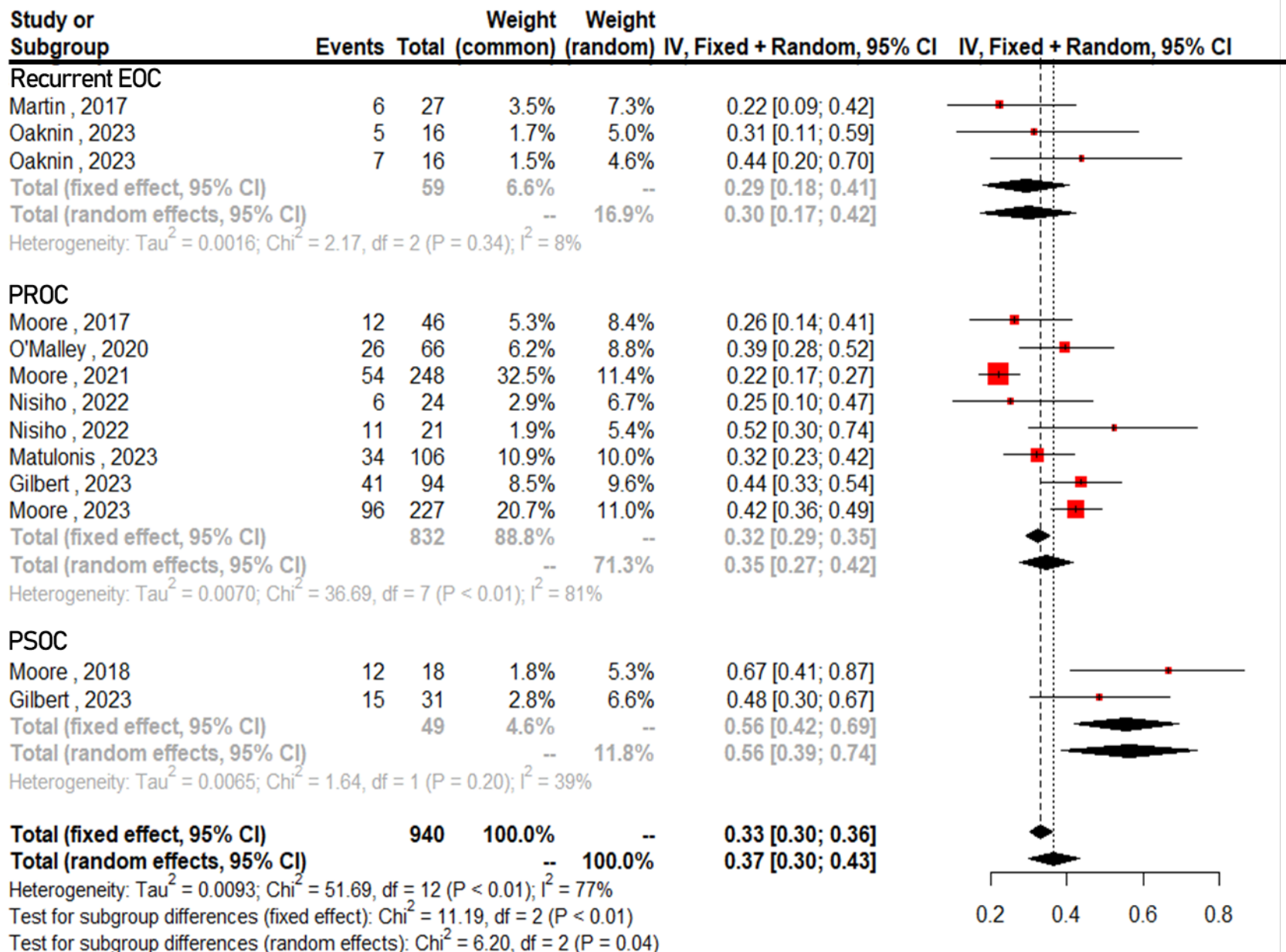

Supplement: Supplementary file 1 — Figure S1. [file CAM4-13-e70392-s004.pdf]

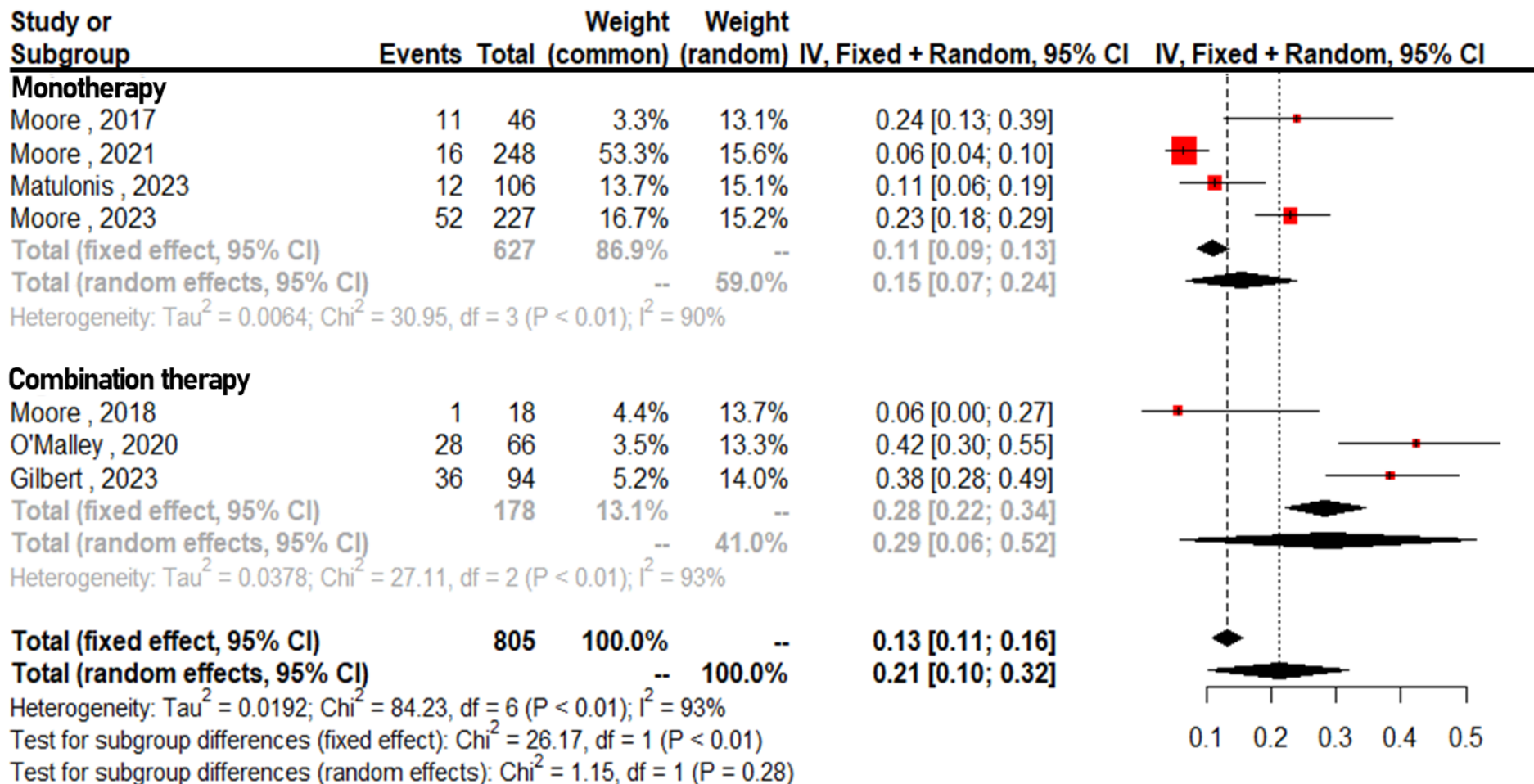

Supplement: Supplementary file 2 — Figure S2. [file CAM4-13-e70392-s001.pdf]

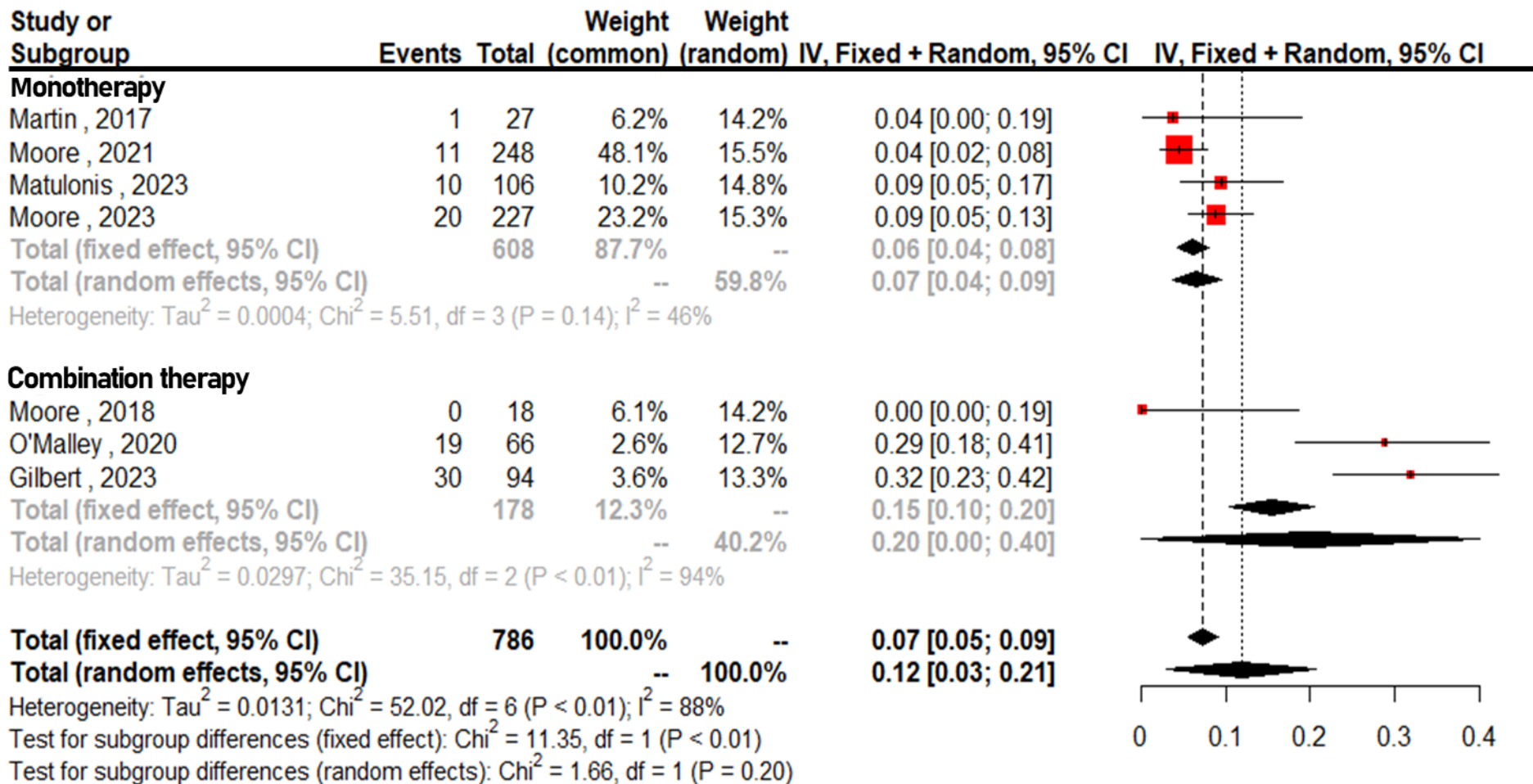

Supplement: Supplementary file 3 — Figure S3. [file CAM4-13-e70392-s005.pdf]

Standard Error

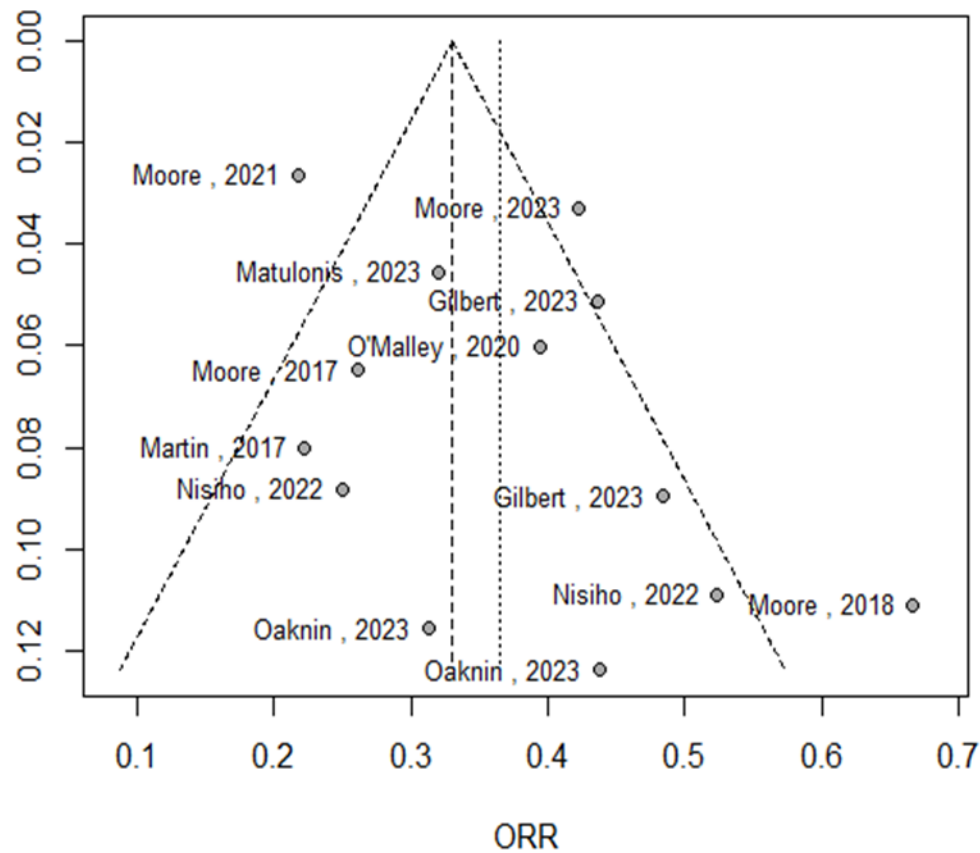

Supplement: Supplementary file 4 — Figure S4. [file CAM4-13-e70392-s002.pdf]
