# Supplementary material for: The Efficacy and Safety of Folate Receptor α‐Targeted Antibody‐Drug Conjugate Therapy in Patients With High‐Grade Epithelial Ovarian, Primary Peritoneal, or Fallopian Tube Cancers: A Systematic Review and Meta‐Analysis
Source: Cancer Med. 2024 Nov 11;13(21):e70392. doi: 10.1002/cam4.70392 (PMC11551784; doi:10.1002/cam4.70392)
Supplement: Supplementary file 5 — Table S1. Table S2. [file CAM4-13-e70392-s003.docx]

**Supplement**

**The efficacy and safety of folate receptor α targeted antibody-drug conjugate therapy in patients with high-grade epithelial ovarian, primary peritoneal, or fallopian tube cancers: A systematic review and meta-analysis**

Eun Taeg Kim, Ji Hyun Kim, Eun Young Park, In Hye Song, Han Song Park, Sang-Yoon Park, Myong Cheol Lim.

* Corresponding author. Email: gynlim@gmail.com

**SUPPLEMENTAL MATERIAL**

**Supplementary Table 1.** Search strategy for systematic review and meta-analysis

**Supplementary Table 2.** Risk of Bias assessment

**Supplementary Figure 1.** Forest plots of ORR according to disease status

**Supplementary Figure 2.** Forest plots of pooled incidence of serious adverse events

**Supplementary Figure 3.** Forest plots of pooled incidence of adverse events leading to drug discontinuation

**Supplementary Figure 4.** Funnel tests for evaluating publication bias

**Supplementary Table 1.** Search strategy for systematic review and meta-analysis

| Number | Search words |
| --- | --- |
| #1 | #1. “FRα” [Title/Abstract] OR “FolRα” [Title/Abstract] OR “FRalpha” [Title/Abstract] OR “folate receptor alpha” [Title/Abstract] OR “folate receptor-alpha” [Title/Abstract] OR “FR” [Title/Abstract] OR “folate receptor” [Title/Abstract] |
| #2 | #2. “ovarian cancer” [Title/Abstract] OR “ovarian carcinoma” [Title/Abstract] OR “ovarian neoplasm” [Title/Abstract] |
|  | Search method |
| #3 | #1 AND #2 |

Database : MEDLINE, EMBASE and the Cochrane Library

Date of Search: Dec 7, 2023

Result: 1119 articles found (MEDLINE : 360, EMBASE : 685, Cochrane Library : 74)

**Supplementary Table 2.** Risk of Bias assessment (MINORS and Risk of Bias 2 (RoB 2))

Methodological index for non-randomized studies (MINORS)

| Non-randomized studies | A stated aim of the study | Inclusion of consecutive patients | Prospective collection of data | End point appropriate to the study aim | Unbiased evaluation of end points | Follow-up period appropriate to the major end point | Loss to follow-up not exceeding 5% | Prospective calculation of the sample size | Total  Scores |
| --- | --- | --- | --- | --- | --- | --- | --- | --- | --- |
| Martin et al 2017 | 2 | 2 | 1 | 2 | 1 | 2 | 1 | 1 | 12 |
| Moore et al 2017 | 2 | 2 | 1 | 2 | 1 | 2 | 1 | 1 | 12 |
| O’Malley et al 2020 | 2 | 2 | 1 | 2 | 1 | 2 | 1 | 1 | 12 |
| Matulonis et al 2023 | 2 | 2 | 1 | 2 | 2 | 2 | 1 | 2 | 14 |
| Gilbert et al 2023 | 2 | 2 | 1 | 2 | 2 | 2 | 1 | 1 | 13 |
| Moore et al 2018 | 2 | 2 | 1 | 2 | 2 | 2 | 1 | 1 | 13 |

Risk of Bias 2 (RoB 2)

| Randomized Studies | Randomization process | Deviations from intended interventions | Missing outcome data | Measurement of the outcome | Selection of reported result | Overall |
| --- | --- | --- | --- | --- | --- | --- |
| Moore et al 2021, FORWARD | Low | Low | Low | Low | Low | Low |
| Moore et al 2023, MIRASOL | Low | Low | Low | Low | Low | Low |
